# Modulation By K^+^ Plus NH_4_^+^ of Microsomal (Na^+^, K^+^)-ATPase Activity in Selected Ontogenetic Stages of the Diadromous River Shrimp *Macrobrachium amazonicum* (Decapoda, Palaemonidae)

**DOI:** 10.1371/journal.pone.0089625

**Published:** 2014-02-21

**Authors:** Francisco A. Leone, Thais M. S. Bezerra, Daniela P. Garçon, Malson N. Lucena, Marcelo R. Pinto, Carlos F. L. Fontes, John C. McNamara

**Affiliations:** 1 Departamento de Química, Faculdade de Filosofia, Ciências e Letras de Ribeirão Preto, Universidade de São Paulo, Ribeirão Preto, SP, Brasil; 2 Departamento de Biologia, Faculdade de Filosofia, Ciências e Letras de Ribeirão Preto, Universidade de São Paulo, Ribeirão Preto, SP, Brasil; 3 Departamento de Biologia Molecular, Centro de Ciências Exatas e da Natureza, Universidade Federal da Paraíba, João Pessoa, PB, Brasil; 4 Departamento de Bioquímica e Imunologia, Faculdade de Medicina de Ribeirão Preto, Universidade de São Paulo, Ribeirão Preto, SP, Brasil; 5 Instituto de Bioquímica Médica, Universidade Federal do Rio de Janeiro, Rio de Janeiro, RJ, Brasil; Albert Einstein College of Medicine, United States of America

## Abstract

We investigate the synergistic stimulation by K^+^ plus NH_4_^+^ of (Na^+^, K^+^)**-**ATPase activity in microsomal preparations of whole zoea I and decapodid III, and in juvenile and adult river shrimp gills. Modulation of (Na^+^, K^+^)-ATPase activity is ontogenetic stage-specific, and particularly distinct between juveniles and adults. Although both gill enzymes exhibit two different sites for K^+^ and NH_4_^+^ binding, in the juvenile enzyme, these two sites are equivalent: binding by both ions results in slightly stimulated activity compared to that of a single ionic species. In the adult enzyme, the sites are not equivalent: when one ion occupies its specific binding site, (Na^+^, K^+^)-ATPase activity is stimulated synergistically by ≈50% on binding of the complementary ion. Immunolocalization reveals the enzyme to be distributed predominantly throughout the intralamellar septum in the gill lamellae of juveniles and adults. Western blot analyses demonstrate a single immunoreactive band, suggesting a single (Na^+^, K^+^)-ATPase α-subunit isoform that is distributed into different density membrane fractions, independently of ontogenetic stage. We propose a model for the modulation by K^+^ and NH_4_^+^ of gill (Na^+^, K^+^)-ATPase activity. These findings suggest that the gill enzyme may be regulated by NH_4_^+^ during ontogenetic development in *M. amazonicum*.

## Introduction

The (Na^+^, K^+^)**-**ATPase is found in the plasma membranes of all animal cells where it is responsible for the asymmetrical, electrogenic, counter transport of Na^+^ and K^+^. It generates membrane resting potential in excitable cells, in addition to establishing ionic gradients that drive various membrane transport processes [1]–[3]. The enzyme belongs to the P_2C_ subfamily of P-type ATPases whose hallmark is the formation of an acyl-phosphate intermediate during the catalytic cycle [4]–[7]. Phosphorylation and dephosphorylation at the D_369_ residue drives the transition between two main conformational changes: E_1_, with a high affinity for intracellular Na^+^, and E_2_ characterized by high affinity for extracellular K^+^ [8]–[12].

The (Na^+^, K^+^)**-**ATPase is an oligomeric protein, and X-ray crystal structure reveals a catalytic α-subunit and a β-subunit, together with an FXYD2 peptide (γ-subunit) [7], [13]–[15]. The α-subunit consists of 10 transmembrane segments and contains the nucleotide binding site, the specific inhibitor binding site, the cation binding sites and the protein kinase phosphorylation domains [6]. The β-subunit is a highly glycosilated, single span, type II membrane protein associated with transmembrane helices αM7 to αM10 [16]. This subunit is required for the correct delivery and assembly of the α-subunit in the plasma membrane, and for occlusion of the K^+^ binding sites [17]. The ≈7,500-Da γ-subunit (FXYD2) is a single-span membrane protein associated with transmembrane helices αM9 and belongs to the FXYD peptide family, a group of small amphiphilic peptides that exhibits the FXYD motif and can regulate pump activity [14], [18], [19]. Exogenous FXYD2 from pig outer renal medulla can activate crab gill (Na^+^, K^+^)**-**ATPase by increasing reaction rate for both regulatory and catalytic ATP sites without affecting ATP affinity [20].

Crustaceans are predominantly marine organisms. Although many are independent of seawater, completing their entire life cycles in fresh water, others may still be in the process of invading fresh water, as suggested by their larval developmental sequence dependent on brackish water, and by their characteristic metabolic, osmotic and ion regulatory mechanisms [21]–[25]. Brackish and freshwater habitats constitute challenging environments since hemolymph osmotic and ionic concentrations are held fairly constant at levels often much higher than the surrounding medium, leading to diffusive ion loss and water gain. Crustaceans inhabiting these media have evolved mechanisms that regulate their hemolymph Na^+^ and Cl^−^ concentrations, both by compensatory ion uptake and by diminishing diffusive ion loss across the gills and other body surfaces [26]–[33].

All crustacean Na^+^-transporting epithelia express the (Na^+^, K^+^)-ATPase, located in basal membrane invaginations, together with basal K^+^ and Cl^−^ channels [33]. However, in strong hyperosmoregulators, the V(H^+^)-ATPase, Na^+^ channels and Cl^−^/HCO^−^_3_ exchangers located in the apical membrane are also key components [34]. Salt uptake models for freshwater palaemonid shrimps like *Macrobrachium amazonicum* propose that active gill Na^+^ absorption ensues through Na^+^ channels in the apical flange membranes of pillar cells in concert with (Na^+^, K^+^)-ATPase activity located in membrane invaginations of the ion-transporting septal cells to which the pillar cells are coupled [32], [35]–[37]. Na^+^ influx is driven by H^+^ extrusion via the apical pillar cell V(H^+^)-ATPase that leads to cellular hyperpolarization, facilitating basal Cl^−^ extrusion [38], [39]. Apical Cl^−^/HCO_3_^−^ exchangers, using HCO_3_^−^ derived from carbonic anhydrase-catalyzed CO_2_ hydration, are thought to transport Cl^−^ into the pillar cell flanges while Cl^−^ efflux proceeds through basal Cl^−^ channel either directly to the hemolymph or to the septal cells. Together with active Na^+^ transport into the hemolymph by the (Na^+^, K^+^)**-**ATPase, K^+^ recycling through septal cell K^+^ channels generates a negative electrical potential that drives Cl^−^ efflux to the hemolymph [31], [32].

The overall success of a species in a given biotope depends on adjustment by each ontogenetic stage to its specific surroundings. In aquatic environments, salt content constitutes the main challenge [40], [41]. While some species spend part of their life cycle in waters where salinity varies little, others migrate between brackish and freshwater biotopes, exposing their semaphoronts to different salinity regimes [25], [40], [42]. In burrowing and burying benthic crustaceans, the high ammonium titers (2–3 mM) characteristic of their silt/sand substrates, may have selected for mechanisms of active ammonium excretion [43]. Further, synergistic stimulation by K^+^ of NH_4_^+^-stimulated (Na^+^, K^+^)-ATPase activity appears to underpin active NH_4_^+^ excretion [44], [45]. This additional (Na^+^, K^+^)-ATPase activity may be advantageous when pumping toxic NH_4_^+^ against its concentration gradient in such environments [46].

Studies of crustacean ontogeny have dealt mainly with marine and estuarine decapods [40]. In contrast, osmoregulatory studies in freshwater Crustacea have focused mostly on adult stages, the larvae having been neglected [22], [40], [47], [48] owing to their small dimensions and reduced hemolymph volume [40], [41], [49]. Fewer studies yet have investigated osmoregulation during early ontogenetic stages [50]–[59]. While the mechanisms of enzyme adjustment to different salinities remain unclear, the role of the (Na^+^, K^+^)-ATPase and other transporters in maintaining hemolymph osmolality and ionic concentration in adult crustaceans is well known [27], [29], [31], [33]. Ontogenetic variations in osmoregulatory ability are associated with changes in salinity tolerance, and involve anatomical changes leading to physiological adjustment, which eventually allows adaptation to biotopes of variable salinity [40], [41], [59].

The Amazon River prawn, *Macrobrachium amazonicum*, is primarily a freshwater species [60]–[62] that migrates to brackish water for spawning, being dependent on brackish water for larval development [24], [63]. It is widely distributed throughout Neotropical South America, inhabiting inland and estuarine waters of the major hydrographic basins, such as the Amazon, Orinoco, São Francisco, Araguaia-Tocantins, Paraná, Paraguay and coastal rivers in north and northeastern Brazil [64], [65]. The adult shrimp is a good hyperosmotic regulator, including excellent chloride regulatory capability, and has been used as a model organism for physiological and molecular studies of salinity tolerance and osmoregulatory mechanisms [24], [39], [49], [62], [63], [66], including larval growth patterns [23], chemical composition [67] and osmoregulatory ability [59], [68].

The life cycle of *M. amazonicum* consists of egg, larval, juvenile, and adult stages, well studied in the natural environment and under aquaculture and laboratory conditions [69]. The early stages of diadromous species like *M. amazonicum* are subject to intense selection pressures that may vary during ontogeny; clearly, knowledge of developmental changes in osmoregulatory ability allows a better understanding of the physiological adjustments that take place during the life cycle [40]. The ontogeny of osmoregulation has been examined in *Macrobrachium petersi* [22], and ontogenetic osmoregulatory ability has been explored in two geographically isolated populations of *M. amazonicum* from different Brazilian biomes [59], [68]. We have kinetically characterized gill (Na^+^, K^+^)**-**ATPase K^+^-phosphatase activity in adult *M. amazonicum* to investigate alterations occurring during reproductive migration into saline water [37].

Recently, we investigated stimulation by ATP, Mg^2+^, Na^+^, K^+^ and NH_4_^+^, separately, and inhibition by ouabain of the (Na^+^, K^+^)**-**ATPase from four ontogenetic stages of *M. amazonicum* [70]. While specific activities differ little, the apparent affinities for ATP and for K^+^ are 2- to 3-fold greater in the decapodid III enzyme; affinity of zoea I (Na^+^, K^+^)-ATPase for Na^+^ is 4-fold less than other stages. Each stage differs considerably in NH_4_^+^ affinity, and in Mg^2+^-stimulated ouabain-insensitive ATPase activity, likely due to ATPases other than the (Na^+^, K^+^)-ATPase, also confirmed by ouabain inhibition kinetics.

Continuing this line of investigation, we now explore the synergistic stimulation by K^+^ plus NH_4_^+^ of (Na^+^, K^+^)**-**ATPase activity in microsomal preparations of whole zoeae I and decapodid III, and in juvenile and adult shrimp gills. We also examine the distribution of (Na^+^, K^+^)**-**ATPase activity in a sucrose density gradient and localization of the enzyme by immunofluorescence labeling in juvenile and adult gill lamellae.

## Materials and Methods

### Material

All solutions were prepared using Millipore MilliQ ultrapure, apyrogenic water. Tris, ATP ditris salt, pyruvate kinase (PK), phosphoenolpyruvate (PEP), NAD^+^, NADH, imidazole, N-(2-hydroxyethyl) piperazine-N19-ethanesulfonic acid (HEPES), lactate dehydrogenase (LDH), ouabain, glyceraldehyde-3-phosphate dehydrogenase (GAPDH), phosphoglycerate kinase (PGK), glyceraldehyde-3-phosphate (G3P), nitroblue tetrazolium (NBT), 5-bromo-4-chloro-3-indole phosphate (BCIP), 4′,6-diamidino-2-phenylindole (DAPI), alamethicin, imidazole, sodium orthovanadate, 3-phosphoglyceraldehyde diethyl acetal, ethacrynic acid, oligomycin, thapsigargin, bafilomycin A_1_ were purchased from the Sigma Chemical Company (Saint Louis, USA). Dimethyl sulfoxide and triethanolamine were from Merck (Darmstadt, Germany). The protease inhibitor cocktail (1 mmol L^−1^ benzamidine, 5 µmol L^−1^ antipain, 5 µmol L^−1^ leupeptin 1 µmol L^−1^, pepstatin A and 5 µmol L^−1^ phenyl-methane-sulfonyl-fluoride) was from Calbiochem (Darmstadt, Germany). Mouse monoclonal antibody IgG α-5 raised against chicken (Na^+^, K^+^)-ATPase α-subunit was from the Development Studies Hybridoma Bank, maintained by the University of Iowa (Iowa, USA). Antimouse IgG, alkaline phosphatase conjugate was purchased from the Promega Corporation (Madison, USA). Optimal Cutting Temperature Compound was from Sakura Tissue-Tek (Torrance, USA). Alexa-fluor 488, donkey anti-mouse IgG, was from Invitrogen (Carlsbad, USA); fluoromount-G and paraformaldehyde were from Electron Microscopy Sciences (Hatfield, USA).

Crystalline suspensions of LDH and PK in 2.9 mol L^−1^ ammonium sulfate (200 µL) were centrifuged at 14,000 rpm for 15 min at 4°C in an Eppendorf Model 5810 refrigerated centrifuge (Hamburg, Germany). The pellet was resuspended in 500 μL of 50 mmol L^−1^ HEPES buffer, pH 7.5, transferred to a YM-10 Microcon filter and washed five times at 10,000 rpm for 15 min at 4°C in the same buffer until complete removal of ammonium ions (tested with the Nessler reagent). Finally, the pellet was resuspended to the original volume. For PGK and GAPDH, the suspension was treated as above with 50 mmol L^−1^ triethanolamine buffer, pH 7.5, containing 1 mmol L^−1^ dithiothreitol. Ammonium sulfate-depleted of PK, LDH, PGK and GAPDH suspensions were used within two days.

G3P was prepared by hydrolysis of 3-phospho-glyceraldehyde diethyl acetal, barium salt, with 150 μL HCl (d = 1.18 g mL^−1^) in a boiling-water bath for 2 min, after removal of the barium salt with Dowex 50H^+^ resin, as recommended by the manufacturer (see Sigma Chem. Co. Product Information for Product Number G5376). Final pH was adjusted to 7.0 with 50 μL triethanolamine just before use. When necessary, enzyme solutions were concentrated on Amicon Ultracell 10K centrifugal filters. All other reagents were of the highest purity commercially available.

### Shrimps

Amazon river shrimps, *Macrobrachium amazonicum*, were produced at the Aquaculture Center, UNESP, Jaboticabal, São Paulo, Brazil from broodstock collected in fresh water at Furo das Marinhas near Santa Bárbara do Pará (1° 13.4500′ S, 48° 17.6320′ W), northeastern Pará State, Brazil, in 2001 [71]. Larval stages were identified according to [72]; zoeae VII, VIII and IX are now termed decapodid I, II and III, respectively, according to the nomenclature proposed by Anger [73].

The stages chosen typify different ontogenetic phases of *M. amazonicum*. Zoea I is a newly-hatched, free-swimming larva that uses internal yolk as an energetic substrate. Decapodid III, the last larval stage, requires brackish water for survival; its yolk reserves have been exhausted and exogenous feeding is necessary [71]. The juvenile is the first benthonic freshwater stage while the adult shrimp are sexually mature.

Zoeae I (≈6000 individuals/preparation, ≈60 µg wet mass) were obtained from hatching tanks (6 ‰ salinity) just after eclosion, guaranteeing that all individuals were in the same stage. The decapodid III stage was obtained from larviculture tanks (12‰ salinity) and individuals were separated under a stereo-microscope using morphological and behavioral characteristics. Groups of decapodid III (≈280 individuals/preparation, ≈650 µg wet mass) were held in aerated carboys containing 32 L water from the larviculture tanks. Juveniles (20 individuals/preparation, ≈700 µg wet gill mass) were collected from freshwater rearing tanks and held in carboys containing 32 L aerated fresh water. Adult male and non-ovigerous female shrimps (20 individuals/preparation, ≈6 g wet gill mass) were collected from freshwater ponds and maintained in carboys containing 32 L aerated pond water.

The various salinities in which the different stages were reared represent those encountered by each ontogenetic stage in its natural environment. To avoid influence of the molting cycle, zoeae I were collected in the evening shortly after hatching. The decapodid III, juvenile and adult stages were used in intermolt, confirmed by stereoscopic microscopy [74]. The individuals in the different stages were transported in their respective carboys to the laboratory and were used immediately for microsomal preparation.

### Preparation of microsomal fractions

For each homogenate prepared, shrimps were anesthetized by chilling on crushed ice immediately before dissection and homogenization. The gills of juvenile and adult shrimps were rapidly dissected, diced and homogenized in a Potter homogenizer in 20 mmol L^−1^ imidazole homogenization buffer, pH 6.8, containing 6 mmol L^−1^ EDTA, 250 mmol L^−1^ sucrose and a protease inhibitor cocktail (20 mL buffer/g wet tissue). For the zoea I and decapodid III stages, whole larvae were homogenized as above. After centrifuging the crude extract at 10,000×g for 35 min at 4°C, the supernatant was placed on crushed ice and the pellet was re-suspended in an equal volume of the homogenization buffer. After further centrifugation as above, the two supernatants were gently pooled and centrifuged at 100,000×g for 90 min at 4°C. The resulting pellet containing the microsomal fraction was homogenized in 20 mmol L^−1^ imidazole buffer, pH 6.8, containing 250 mmol L^−1^ sucrose (15 mL buffer/g wet tissue). Finally, 0.5-mL aliquots were rapidly frozen in liquid nitrogen and stored at −20°C. No appreciable loss of (Na^+^, K^+^)-ATPase activity was seen after two-month's storage of the microsomal enzyme prepared either from whole larvae or gill tissue. When required, the aliquots were thawed, placed on crushed ice and used immediately.

### Measurement of ATP hydrolysis

Total ATPase activity was assayed at 25°C using a PK/LDH coupling system [75] in which ATP hydrolysis was coupled to NADH oxidation according to [70]. The oxidation of NADH was monitored at 340 nm (ε_340 nm_, _pH 7.5_ = 6,200 mol^−1^ L cm^−1^) in a Hitachi U-3000 spectrophotometer equipped with thermostatted cell holders. Standard conditions were: 50 mmol L^−1^ HEPES buffer, pH 7.5, 2 mmol L^−1^ ATP, containing 5 mmol L^−1^ MgCl_2_, 20 mmol L^−1^ KCl, 0.14 mmol L^−1^ NADH, 2 mmol L^−1^ PEP, 82 µg PK (49 U), 110 µg LDH (94 U), and 50 mmol L^−1^ NaCl (for zoea I and juveniles) or 20 mmol L^−1^ NaCl (for decapodid III and adults), in a final volume of 1 mL. Alternatively, ATPase activity was estimated using a GAPDH/PGK linked system coupled to the reduction of NAD^+^ at 340 nm [70]. Standard conditions were: 50 mmol L^−1^ triethanolamine buffer, pH 7.5, 2 mmol L^−1^ ATP, containing 5 mmol L^−1^ MgCl_2_, 20 mmol L^−1^ KCl, 1 mmol L^−1^ NAD^+^, 0.5 mmol L^−1^ sodium phosphate, 1 mmol L^−1^ G3P, 150 μg GAPDH (12 U), 20 μg PGK (9 U), and 50 mmol L^−1^ NaCl (for zoea I and juveniles) or 20 mmol L^−1^ NaCl (for decapodid III and adults) in a final volume of 1 mL. The two coupling systems gave equivalent results with a difference of less than 10%.

ATP hydrolysis was also estimated with 3 mmol L^−1^ ouabain to assess ouabain-insensitive activity. The difference in activity measured in the absence (total ATPase activity) or presence of ouabain (ouabain-insensitive activity) represents the (Na^+^, K^+^)-ATPase activity. The effect of various inhibitors on total ATPase activity was examined as above, preincubating the enzyme at 25°C for 10 min with each inhibitor. Thapsigargin and bafilomycin were prepared in DMSO, oligomycin and aurovertin in ethanol, and ethacrynic acid and theophylline in distilled water.

ATP hydrolysis was also estimated at 25°C after 10 min pre-incubation with alamethicin (1 mg/mg protein) to demonstrate the presence of leaky and/or disrupted vesicles. Controls without added enzyme were included in each experiment to quantify the non-enzymatic hydrolysis of substrate. Initial velocities were constant for at least 15 min provided that less than 5% of the total NADH (or NAD^+^) was oxidized (or reduced). The reaction rate for each modulator was estimated in duplicate using identical aliquots from the same preparation. Mean values were used to fit each corresponding saturation curve, which was repeated three times utilizing different microsomal homogenates (N = 3). One enzyme unit (U) is defined as the amount of enzyme that hydrolyzes 1.0 nmol of ATP per minute, at 25°C, and (Na^+^, K^+^)-ATPase specific activity is given as nmol Pi min^−1^ mg^−1^ total protein.

### Western blot analysis

SDS-PAGE of the gill microsomes from shrimps held in fresh water were performed as described by [76] using 4 µg and 160 µg protein/slot for protein staining and blotting analysis, respectively. After electrophoresis, the gel was split, one half being stained with silver nitrate and the other electroblotted using a Gibco BRL Mini-V 8–10 system (Gaithersburg, USA) employing a nitrocellulose membrane according to [77]. The nitrocellulose membrane was blocked for 10 h with 5% nonfat dry milk freshly prepared in 50 mmol L^−1^ Tris.HCl buffer, pH 8.0, containing 150 mmol L^−1^ NaCl and 0.1% Tween 20, with constant agitation. The membrane was incubated for 30 min at 25°C in a 1∶10 dilution (2.1 µg mL^−1^) of the α-5 monoclonal antibody. After washing three times in 50 mmol L^−1^ Tris.HCl buffer, pH 8.0, containing 150 mmol L^−1^ NaCl and 0.1% Tween 20, the membrane was incubated for 30 min at 25°C with an anti-mouse IgG, alkaline phosphatase conjugate, diluted 1∶7,500. The membrane was washed three times in 50 mmol L^−1^ Tris.HCl buffer, pH 8.0, containing 150 mmol L^−1^ NaCl and 0.1% Tween 20, and specific antibody binding was developed in 100 mmol L^−1^ Tris.HCl buffer, pH 9.5, containing 100 mmol L^−1^ NaCl, 5 mmol L^−1^ MgCl_2_, 0.2 mmol L^−1^ NBT and 0.8 mmol L^−1^ BCIP. Controls consisting of membranes incubated with the secondary antibody without previous incubation with the α-5 antibody were included in each experiment. Western blot analysis for each experiment was repeated three times using different tissue preparations from separate pools of 15–30 shrimps each. Immunoblots were scanned and imported as JPG files into a commercial software package (Kodak 1D 3.6) where immuno-reaction densities were quantified and compared.

### Immunolocalization of the gill (Na^+^, K^+^)-ATPase

Fourth, right side gills were dissected and incubated in a fixative solution containing 2% *p*-formaldehyde in a phosphate buffered saline, PBS, (Na_2_HPO_4_ 10 mmol L^−1^, KH_2_PO_4_ 2 mmol L^−1^, NaCl 137 mmol L^−1^, KCl 2.7 mmol L^−1^, 290 mOsm kg^−1^ H_2_O), pH 7.4, for 1 h, then embedded in Optimal Cutting Temperature Compound. 10-µm thick cryosections were taken transversely to the gill lamella long-axis using a Microm HM 505E model Cryostat Microtome (Walldorf, Germany) at −25°C and collected on gelatin-coated slides (Bloom 225). Cryosections were preincubated for 20 min with 100 mmol L^−1^ glycine in phosphate buffered saline (PBS) to mask free aldehyde groups and were incubated for 10 min in blocking solution containing 1% bovine serum albumin and 0.1% gelatin in PBS.

(Na^+^, K^+^)-ATPase immunolocalization was performed using a mouse monoclonal IgG α-5 antibody raised against chicken (Na^+^, K^+^)-ATPase α-subunit [78]. Droplets of primary antibody, diluted to 20 mg ml^−1^ in PBS (1∶1.75) were placed over the sections, which were incubated for 1 h at room temperature in a humid chamber. Negative control sections were incubated in blocking solution without the primary antibody. After washing six times for 5 min each in blocking solution to remove unbound antibodies, the sections were incubated for 45 min in droplets of a donkey anti-mouse IgG secondary antibody conjugated with Alexa-fluor 488 diluted 1∶450 in PBS, and then rinsed six times for 5 min each in PBS. To locate nuclei, sections were stained for 20 min with DAPI, diluted 1∶200 in PBS.

Sections were mounted in Fluoromount-G slide-mounting medium on Knittel Starfrost slides with cover slips (Bielefeld, Germany). They were observed and photographed using an Olympus BX-50 fluorescence microscope (Olympus America Inc., Melville, NY) equipped with a SPOT RT3 25.4 2 Mb Slider camera (SPOT Imaging Solutions Inc., Sterling Heights, MI, USA) employing differential interference contrast microscopy and excitation/emission wavelengths of 358/461 nm (DAPI) and 495/519 nm (Alexa-fluor 488).

### Protein measurement

Protein concentration was estimated using the Coomassie Blue G dye-binding assay [79] employing bovine serum albumin as the standard.

### Estimation of kinetic parameters

The kinetic parameters V_M_ (maximum velocity), K_0.5_ (apparent dissociation constant), K_M_ (Michaelis-Menten constant) and the n_H_ (Hill coefficient) value for ATP hydrolysis under the different assay conditions were calculated using SigrafW software [80], freely available from http://portal.ffclrp.usp.br/sites/fdaleone/downloads. The kinetic parameters furnished in the tables are calculated values and represent the mean (± SEM) also derived from three (N = 3) microsomal preparations. Data were analyzed using a one-way analysis of variance (inhibitor) followed by Student-Newman-Keuls multiple means testing. Effects and differences were considered significant at P = 0.05.

## Results

### Continuous-density sucrose gradient centrifugation analysis

**Figure 1:**
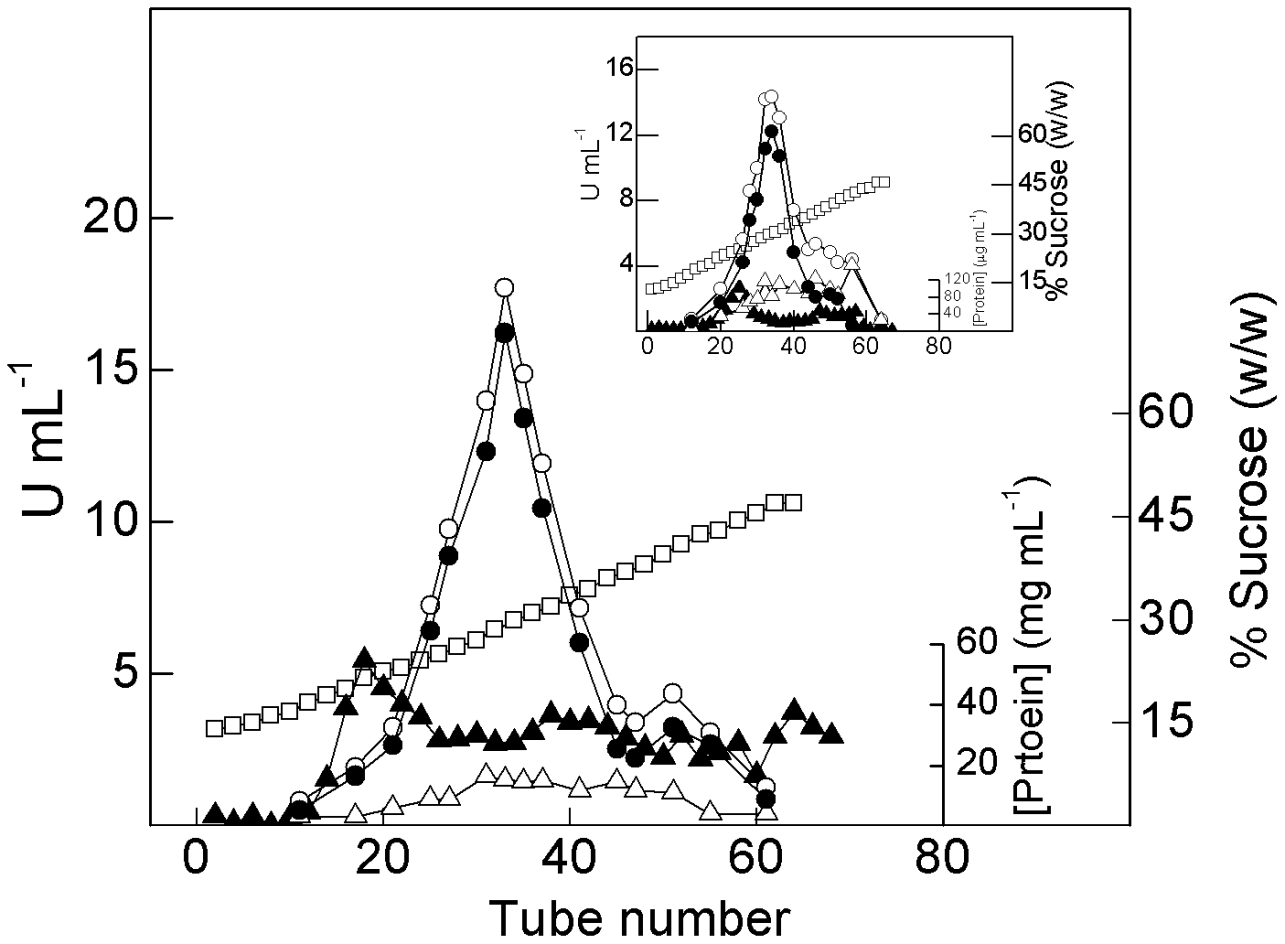
Sucrose density gradient centrifugation of a microsomal fraction from gill tissue of juvenile and adult *M. amazonicum*. Aliquots containing 4.5% (w/w) continuous sucrose density gradients. Fractions (0.5 mL) collected from the bottom of each gradient were analyzed for total ATPase activity (○), (Na^+^, K^+^)-ATPase activity (•), ouabain-insensitive ATPase activity (▵), protein concentration (▴) and sucrose concentration (□). **Inset**: Adult gill tissue.

The distributions along the continuous-density sucrose gradient of gill microsomal (Na^+^, K^+^)-ATPase activity from juvenile and adult *M. amazonicum* is shown in Fig. 1. In the juvenile two protein peaks were identified: a main peak between 23 to 36% of sucrose exhibiting maximum activity of 16.2 U mL^−1^, and a lesser heavier peak that sediments between 38 and 44% sucrose showing a maximum activity of 3.2 U mL^−1^. Ouabain-insensitive ATPase activities, corresponding to ≈12% and ≈24% of peak I and II total ATPase activities, respectively, suggest the presence of ATPases other than the (Na^+^, K^+^)-ATPase. Adult gills also showed two protein peaks (inset to Fig. 1). The main peak showed a maximum (Na^+^, K^+^)-ATPase activity of 12.5 U mL^−1^ and sedimented between 26 and 34% sucrose; up to 85% of this activity was inhibited by 3 mmol L^−1^ ouabain. No detectable (Na^+^, K^+^)-ATPase activity was seen in the minor heavier protein peak which sedimented between 36 and 42% sucrose.

### SDS-PAGE and Western Blot analyses

**Figure 2:**
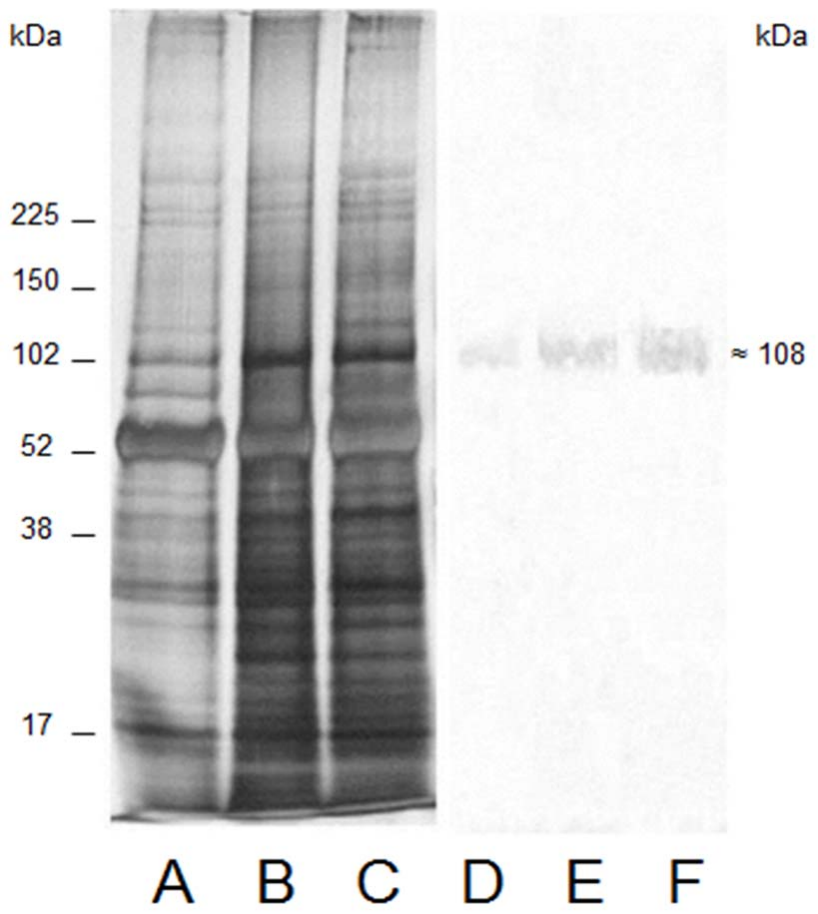
SDS-PAGE and Western blot analyses of microsomal fractions from whole decapodid III, and juvenile and adult *M. amazonicum* gills. Electrophoresis was performed in a 5–20% polyacrylamide gel using 4 μg microsomal protein for silver staining and 160 μg for Western blotting. The analysis was repeated three times (*N* = 3) using aliquots from different homogenates prepared from each ontogenetic stage. **Silver nitrate-stained gels**: **A-** Decapodid III. **B-** Juvenile. **C**- Adult. **Western blots**: **D-** Decapodid III. **E**- Juvenile. **F**- Adult.

SDS-PAGE and Western blot analyses of microsomal preparations of whole decapodid III, and juvenile and adult *M. amazonicum* gills are compared in Fig. 2. The silver stained gels demonstrate that the microsomal fraction of whole decapodid III homogenate (lane A) exhibits a protein profile similar to those of juvenile (lane B) and adult (lane C) gill homogenates. The Western blot analysis reveals that the single immunoreactive bands for the decapodid III (lane D), juvenile (lane E) and adult (lane F) stages correspond to the (Na^+^, K^+^)-ATPase α-subunit with a molecular mass of ≈108 kDa.

### Immunolocalization of the gill (Na^+^, K^+^)-ATPase α-subunit

**Figure 3:**
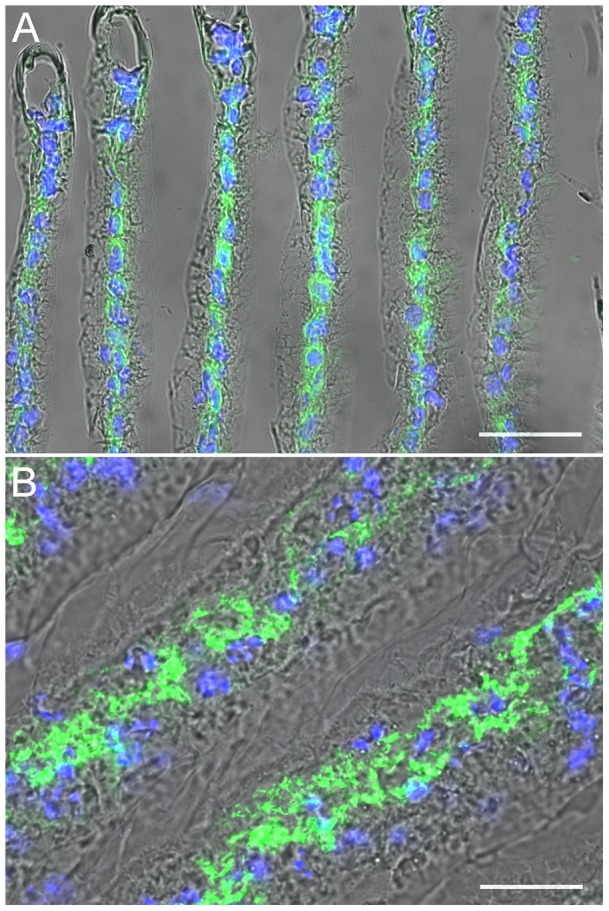
Immunolocalization of the gill (Na^+^, K^+^)-ATPase α-subunit in juvenile and adult *M. amazonicum.* Frozen cross sections taken transversely to the gill lamellae long axes were incubated with mouse monoclonal IgG α-5 antibody raised against chicken (Na^+^, K^+^)-ATPase α-subunit then incubated in donkey anti-mouse IgG secondary antibody conjugated with Alexa-fluor 488. Phase contrast/DAPI/α-5 images demonstrating typical lamellar structure. Immunofluorescence labeling (Alexa-fluor 488, 495/519 nm) showing distribution of the (Na^+^, K^+^)-ATPase α-subunit (green) located predominantly in the intralamellar septal cells identified by their DAPI-stained nuclei (blue). **A-** Juvenile gill lamellae. **B**- Adult gill lamellae. Scale bars  = 50 µm.

Immunolocalization of the (Na^+^, K^+^)-ATPase α-subunit in juvenile and adult *M. amazonicum* gills revealed positive immunolabeling located mainly along the intralamellar septum in both juvenile (Fig. 3A) and adult (Fig. 3B) gill lamellae. DAPI-stained nuclei clearly revealed the intralamellar septum and abutting pillar cell bases. Although individual septal cells were often not discernible, labeling was not present in the pillar cells underlying the cuticle. Control sections without primary antibody showed no signal (not shown).

### Effect of NH_4_^+^ on K^+^ stimulation of zoea I and decapodid III (Na^+^, K^+^)-ATPase activity

**Figure 4:**
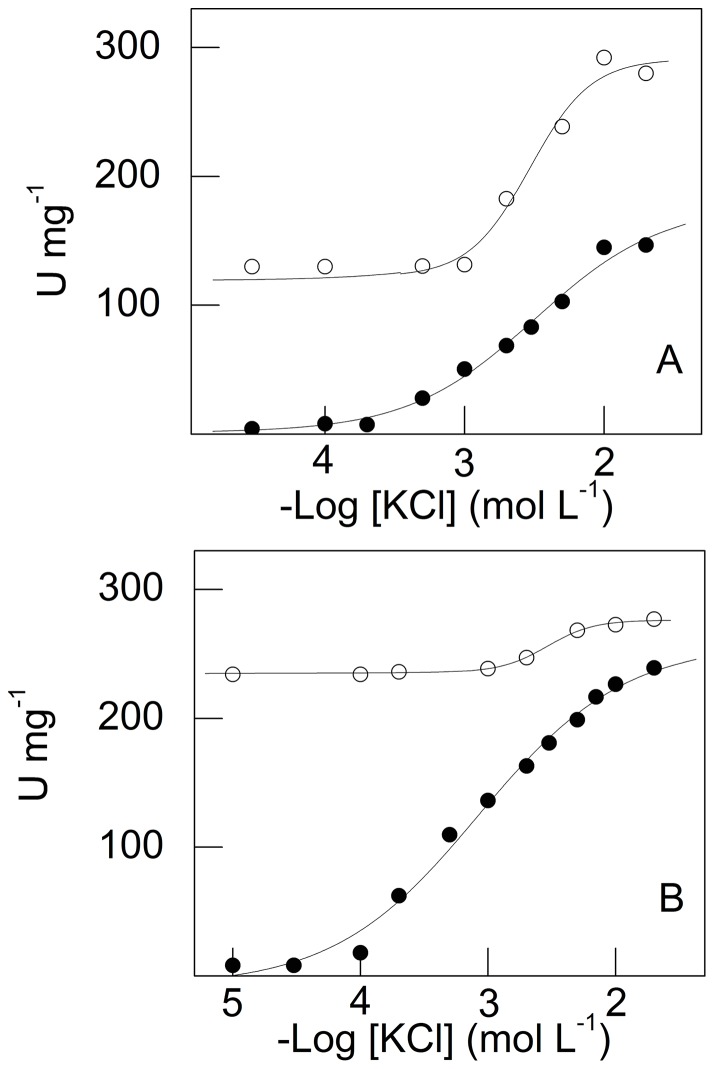
Effect of NH_4_^+^ concentration on modulation by K^+^ of microsomal (Na^+^, K^+^)-ATPase activity in whole *M. amazonicum* zoea I and decapodid III. Data are the mean ± SEM (*N* = 3) obtained using duplicate aliquots containing 13.4 μg protein (zoea I) and 7.2 μg protein (decapodid III) from three different homogenates. Activity was assayed at 25°C in 50 mmol L^−1^ triethanolamine buffer (pH 7.5), containing 2 mmol L^−1^ ATP, 5 mmol L^−1^ MgCl_2_, 1.0 mmol L^−1^ NAD^+^, 0.5 mmol L^−1^ sodium phosphate, 1.0 mmol L^−1^ G3P, 150 μg GAPDH (12 U), 20 μg PGK (9 U) and NaCl (50 mmol L^−1^ for zoea I and 20 mmol L^−1^ for decapodid III) in a final volume of 1 mL. **A-** Zoea I. **B-** Decapodid III. NH_4_^+^ concentration: (•) none, (○) 30 mmol L^−1^.

The effect of NH_4_^+^ on K^+^-stimulated (Na^+^, K^+^)-ATPase activity in homogenates of whole zoea I and decapodid III is shown in Fig. 4. Under saturating ATP (2 mmol L^−1^), Na^+^ (50 mmol L^−1^ for zoea I, 20 mmol L^−1^ for decapodid III) and Mg^2+^ (5 mmol L^−1^) concentrations, and in the absence of NH_4_^+^, stimulation of (Na^+^, K^+^)-ATPase activity in zoea I by K^+^ (from 5×10^−5^ mol L^−1^ to 2×10^−2^ mol L^−1^) reaches a maximum of V_M_ = 150.5±7.3 nmol Pi min^−1^ mg^−1^ with K_0.5_ = 3.2±0.2 mmol L^−1^ (Fig. 4A and Table 1). With 30 mmol L^−1^ NH_4_^+^, the maximum rate was 289.9±5.8 nmol Pi min^−1^ mg^−1^, showing cooperative kinetics, with K_0.5_ = 3.2±0.3 mmol L^−1^. Although stimulation with NH_4_^+^ plus K^+^ reached 92%, K_0.5_ was unchanged compared to that without NH_4_^+^ (Table 1). In the absence of NH_4_^+^, substrate hydrolysis obeyed Michaelis-Menten kinetics, but with NH_4_^+^, kinetics was cooperative. The effect of NH_4_^+^ on K^+^ stimulation of (Na^+^, K^+^)-ATPase activity in decapodid III also follows Michaelis-Menten kinetics, reaching a maximum rate of 247.0±10.5 nmol Pi min^−1^ mg^−1^ with K_0.5_ = 0.9±0.1 mmol L^−1^ as K^+^ increases from 10^−5^ mol L^−1^ to 2×10^−2^ mol L^−1^ (Fig. 4B). With 30 mmol L^−1^ NH_4_^+^, (Na^+^, K^+^)-ATPase activity was stimulated to maximum rate of 275.6±3.7 nmol Pi min^−1^ mg^−1^ with K_0.5_ = 3.1±0.2 mmol L^−1^ as the enzyme becomes fully saturated with K^+^. Synergistic stimulation of (Na^+^, K^+^)-ATPase activity (≈12%) concomitant with a ≈4-fold increase in K_0.5_ occurred with NH_4_^+^ plus K^+^, obeying cooperative kinetics (Table 1).

**Table 1:** Kinetic parameters for the stimulation by K^+^ and NH_4_^+^ of (Na^+^, K^+^)-ATPase activity in whole zoea I and decapodid III, and juvenile and adult *M. amazonicum* gills.

[K^+^] (mmol L^−1^)	[NH_4_^+^] (mmol L^−1^)	V_M_ (nmol Pi min^−1^ mg^−1^)		K_M_ or K_0.5_ (mmol L^−1^)		n_H_		V_M_/K_0.5_×10^6^	
		Juvenile	Adult	Juvenile	Adult	Juvenile	Adult	Juvenile	Adult
0.01 to 50	0	178.4±3.3	169.9±0.7	1.3±0.1	1.0±0.2	1.0	1.6	138	170
0.01 to 50	0.3	203.8±1.2	-	1.0±0.1	-	1.3	-	198	-
0.01 to 50	1	206.0±0.8	-	3.9±0.7	-	2.1	-	53	-
0.01 to 50	2	-	200.3±1.6	-	1.6±0.2	-	1.0	-	124
0.01 to 50	3	-	219.7±1.6	-	1.6±0.2	-	1.0	-	137
0.01 to 50	5	204.5±1.6	230.9±2.0	2.7±0.2	2.3±0.2	2.8	1.4	76	101
0.01 to 50	10	203.6±1.6	250.1±2.0	2.5±0.2	3.8±0.2	2.1	1.3	81	65
0.01 to 50	30	205.4±2.5	-	-	-	-	-	-	-
0	1 to 100	205.9±2.2	193.4±1.4	1.9±0.2	4.8±0.3	1.0	1.8	110	41
0.4	1 to 100	207.1±1.3	-	1.1±0.2	-	1.2	-	195	-
0.5	1 to 100	-	214.1±1.6	-	4.0±0.2	-	2.1	-	54
2	1 to 100	202.7±0.9	215.4±1.1	0.9±0.1	2.1±0.2	1.7	2.0	225	101
5	1 to 100	219.7±1.0	240.1±4.7	1.6±0.2	1.6±0.2	1.5	2.2	138	148
10	1 to 100	218.1±0.7	-	0.8±0.2	-	1.3	-	283	-
20	1 to 100	217.5±1.2	263.4±8.8	-	1.4±0.2	-	2.2	-	188

		Zoea I	Decapodid III	Zoea I	Decapodid III	Zoea I	Decapodid III	Zoea I	Decapodid III
0.01 to 20	0	150.5±7.3	247.0±10.5	3.2±0.2	0.9±0.1	1.0	1.0	47	274
0.01 to 20	30	289.9±5.8	275.6±3.7	3.2±0.3	3.1±0.2	2.2	2.8	91	89
0	1 to 100	271.9±3.1	273.9±8.0	6.3±0.4	3.1±0.2	3.4	3.3	43	88
20	1 to 100	280.1±7.3	271.1±7.2	7.7±0.7	5.7±0.4	1.3	2.0	36	48

Initial rates were measured in 50 mmol L^−1^ HEPES buffer, pH 7.5, containing 2 mmol L^−1^ ATP, 5 mmol L^−1^ MgCl_2_, 50 mmol L^−1^ NaCl, and the given concentrations of KCl and NH_4_Cl, in a final volume of 1.0 mL. Data are the mean ± SD from at least three different larval or gill preparations.

### Effect of NH_4_^+^ on K^+^ stimulation of juvenile and adult gill (Na^+^, K^+^)-ATPase activity

**Figure 5:**
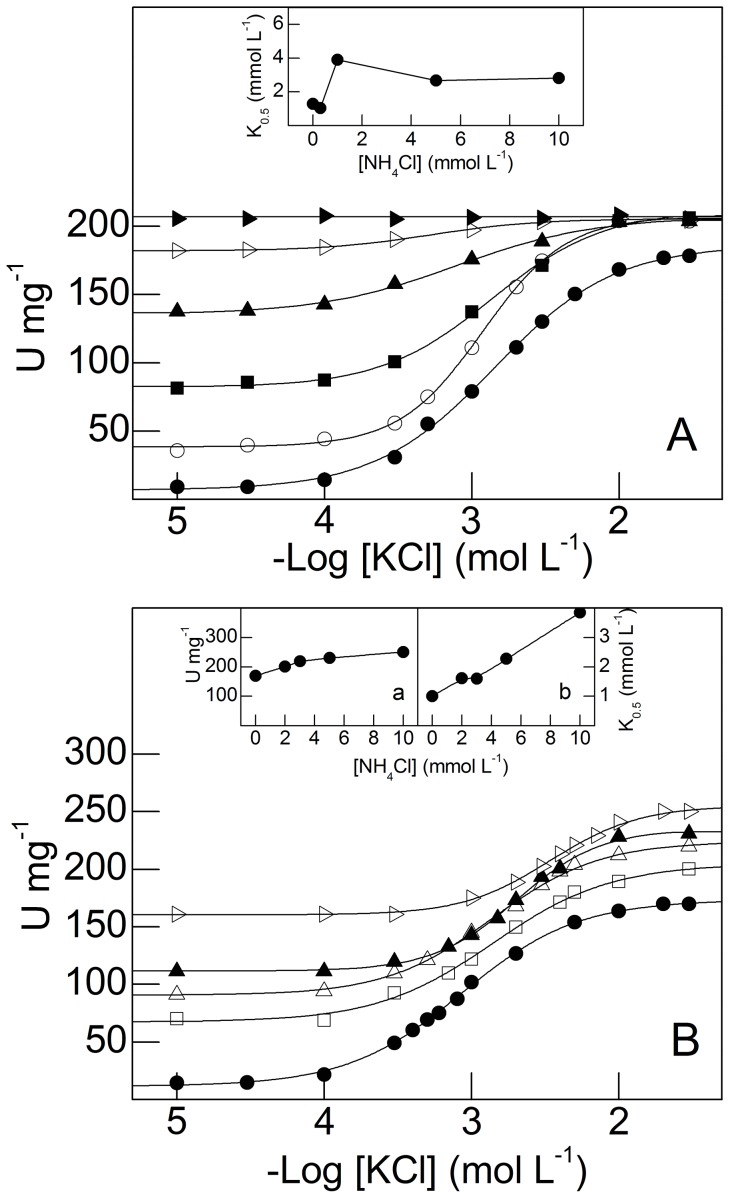
Effect of NH_4_^+^ concentration on modulation by K^+^ of microsomal (Na^+^, K^+^)-ATPase activity in gill tissue from juvenile and adult *M. amazonicum*. Data are the mean ± SEM (*N* = 3) obtained using duplicate aliquots containing 9.5 μg protein (juveniles) and 10.7 μg protein (adults) from three different gill homogenates. Activity was assayed at 25°C in 50 mmol L^−1^ triethanolamine buffer (pH 7.5), containing 2 mmol L^−1^ ATP, 5 mmol L^−1^ MgCl_2_, 1.0 mmol L^−1^ NAD^+^, 0.5 mmol L^−1^ sodium phosphate, 1.0 mmol L^−1^ G3P, 150 μg GAPDH (12 U), 20 μg PGK (9 U) and NaCl (50 mmol L^−1^ for juveniles and 20 mmol L^−1^ for adults), in a final volume of 1 mL. **A-** Juveniles. **Inse**t: Variation in K_0.5_ with NH_4_^+^ concentration. **B-** Adults. Variation in V_M_ (inset **a**) and K_0.5_ (inset **b**) with NH_4_^+^ concentration. NH_4_^+^ concentration: (•) none, (○) 0.3 mmol L^−1^, (▪) 1 mmol L^−1^, (□) 2 mmol L^−1^, (▵)3 mmol L^−1^, (▴) 5 mmol L^−1^, (▹)10 mmol L^−1^, (▸) 30 mmol L^−1^.

The effect of NH_4_^+^ on K^+^-stimulated gill (Na^+^, K^+^)-ATPase activity in juvenile and adult *M. amazonicum* is shown in Fig. 5. Under saturating ATP (2 mmol L^−1^), Na^+^ (50 mmol L^−1^) and Mg^2+^ (5 mmol L^−1^) concentrations, and without NH_4_^+^, stimulation of gill microsomal (Na^+^, K^+^)-ATPase activity of juveniles by K^+^ (from 10^−5^ mol L^−1^ to 5×10^−2^ mol L^−1^) reached a maximum rate of 178.4±3.3 nmol Pi min^−1^ mg^−1^ with K_0.5_ = 1.3±0.1 mmol L^−1^, obeying Michaelis-Menten kinetics (Fig. 5A and Table 1). Modulation by K^+^ of (Na^+^, K^+^)-ATPase activity at fixed NH_4_^+^ concentrations (0.3 mmol L^−1^ to 30 mmol L^−1^) resulted in minor stimulation (≈15%), reaching a maximum rate of 205.4±2.5 nmol Pi min^−1^ mg^−1^, at 30 mmol L^−1^ NH_4_^+^, with little change in K_0.5_ values (inset to Fig. 5A and Table 1). Despite the slight stimulation by K^+^ plus NH_4_^+^, the convergence of the activity curves to similar maximum rates is remarkable. These findings suggest that NH_4_^+^ and K^+^ bind to separate but equivalent sites on the enzyme molecule, each ion modulating the activity of the other. There is no significant synergistic modulation by NH_4_^+^ of K^+^-stimulated ATPase gill activity in *M. amazonicum* juveniles.

Gill (Na^+^, K^+^)-ATPase activity in adult *M. amazonicum* showed a different stimulation pattern (Fig. 5B). Under saturating ATP (2 mmol L^−1^), Na^+^ (20 mmol L^−1^) and Mg^2+^ (5 mmol L^−1^) concentrations, and without NH_4_^+^, stimulation by K^+^ (from 10^−5^ mol L^−1^ to 5×10^−2^ mol L^−1^) reached a maximum rate of 169.9±0.7 nmol Pi min^−1^ mg^−1^ with K_0.5_ = 1.0±0.2 mmol L^−1^, following cooperative kinetics (Table 1). Synergistic stimulation by K^+^ of (Na^+^, K^+^)-ATPase activity (47%) was seen at different NH_4_^+^ concentrations, resulting in maximum rate of 250.1±2.0 nmol Pi min^−1^ mg^−1^ and K_0.5_ = 3.8±0.2 mmol L^−1^ (inset a to Fig. 5B), likely due to both NH_4_^+^ and K^+^ binding to different sites on the enzyme molecule. Stimulation resulted in a 4-fold increase in K_0.5_ (inset b to Fig. 5B).

### Effect of K^+^ on NH_4_^+^ stimulation of zoea I and decapodid III (Na^+^, K^+^)-ATPase activity

**Figure 6:**
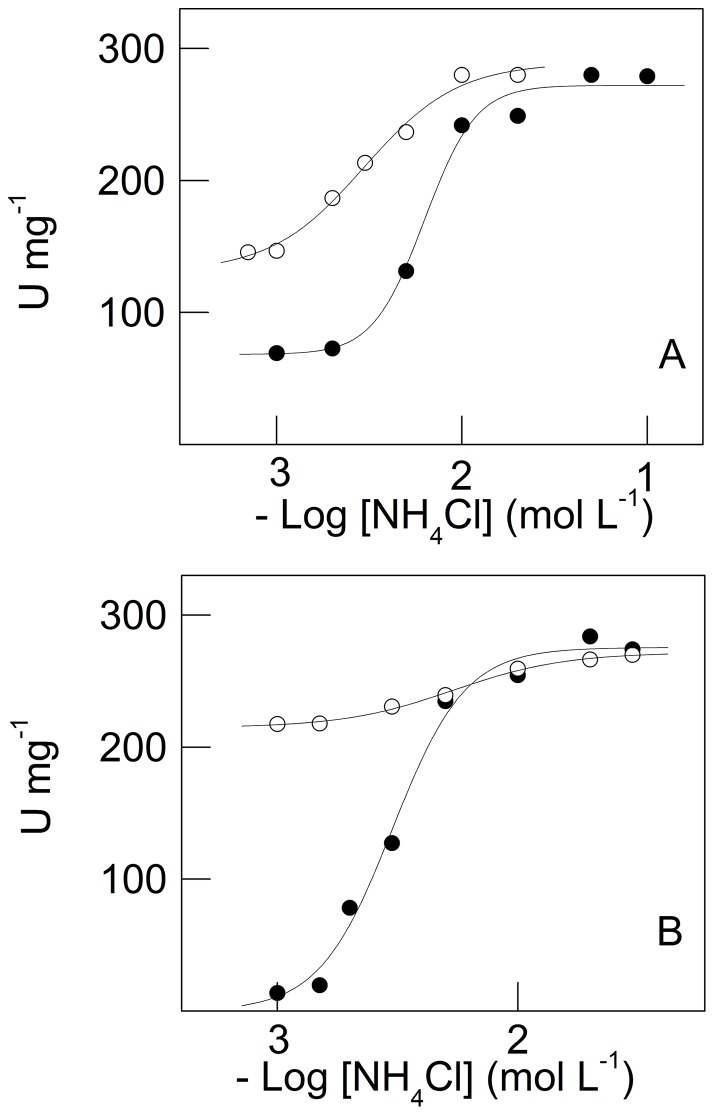
Effect of K^+^ concentration on modulation by NH_4_^+^ of microsomal (Na^+^, K^+^)-ATPase activity in whole *M. amazonicum* zoea I and decapodid III. Data are the mean ± SEM (*N* = 3) obtained using duplicate aliquots containing 13.4 μg protein (zoea I) and 7.2 μg protein (decapodid III) from three different homogenates. Activity was assayed at 25°C in 50 mmol L^−1^ triethanolamine buffer (pH 7.5), containing 2 mmol L^−1^ ATP, 5 mmol L^−1^ MgCl_2_, 1.0 mmol L^−1^ NAD^+^, 0.5 mmol L^−1^ sodium phosphate, 1.0 mmol L^−1^ G3P, 150 μg GAPDH (12 U), 20 μg PGK (9 U) and NaCl (50 mmol L^−1^ for juveniles and 20 mmol L^−1^ for adults), in a final volume of 1 mL. A- Zoea I. B- Decapodid III. K^+^ concentration (•) none, (○) 20 mmol L^−1^ K^+^.

The effect of K^+^ on NH_4_^+^ stimulation of whole zoea I and decapodid III (Na^+^, K^+^)-ATPase activity is shown in Fig. 6. Under saturating ATP (2 mmol L^−1^), Na^+^ (50 mmol L^−1^ and 20 mmol L^−1^ for zoea I and decapodid III, respectively) and Mg^2+^ (5 mmol L^−1^) concentrations, without K^+^, stimulation of (Na^+^, K^+^)-ATPase activity in zoea I by NH_4_^+^ (from 10^−3^ mol L^−1^ to 10^−1^ mol L^−1^) was maximum at 271.9±3.1 nmol Pi min^−1^ mg^−1^ with K_0.5_ = 6.3±0.4 mmol L^−1^ (Fig. 6A and Table 1); site-site interactions were observed (n_H_ = 3.4). At 20 mmol L^−1^ K^+^, stimulation was negligible (V_M_ = 280.1±7.3 nmol Pi min^−1^ mg^−1^) with a slight increase in K_0.5_ (7.7±0.7 mmol L^−1^). In decapodid III, K^+^ also modulated NH_4_^+^ stimulated (from 10^−3^ mol L^−1^ to 7×10^−2^ mol L^−1^) (Na^+^, K^+^)-ATPase activity obeying cooperative kinetics. While K_0.5_ increased almost 2-fold, V_M_ was unaffected (Fig. 6B and Table 1).

### Effect of K^+^ on NH_4_^+^ stimulation of juvenile and adult gill (Na^+^, K^+^)-ATPase activity

**Figure 7:**
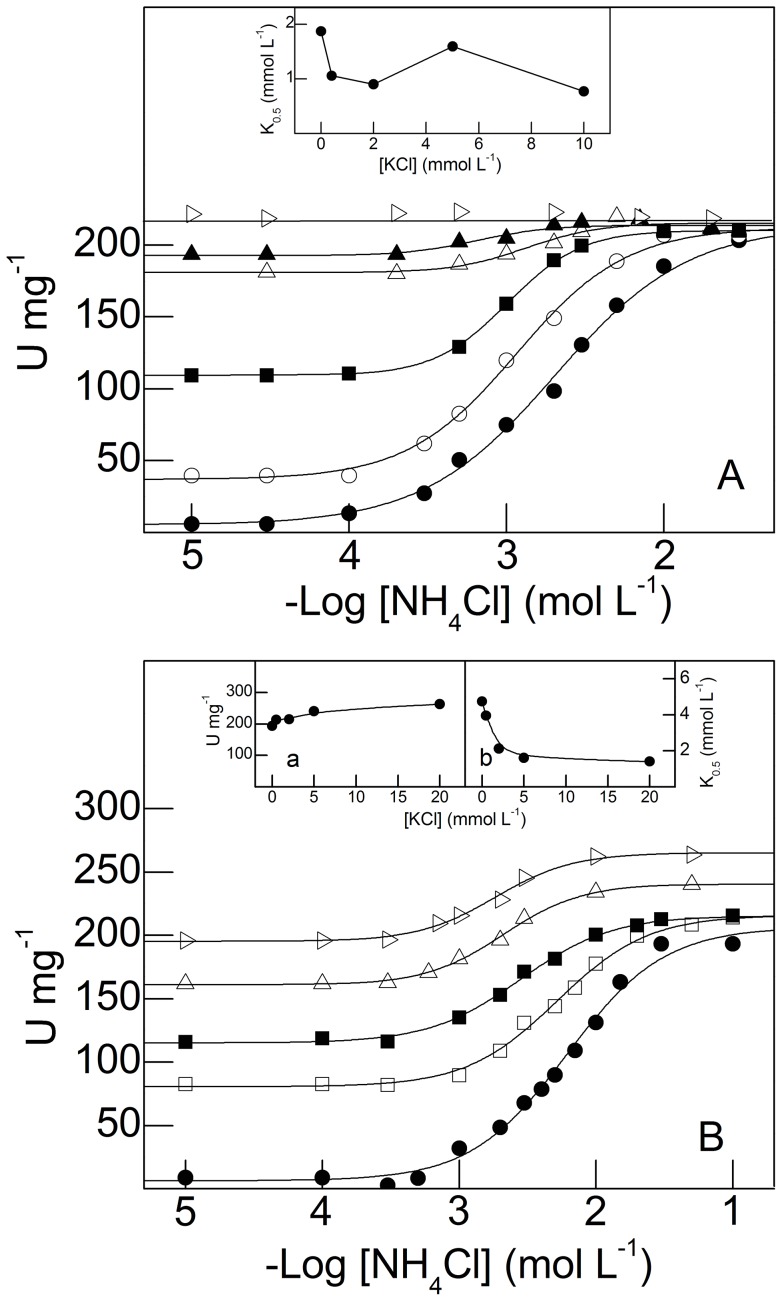
Effect of K^+^ concentration on modulation by NH_4_^+^ of microsomal (Na^+^, K^+^)-ATPase activity in gill tissue from juvenile and adult *M. amazonicum*. Data are the mean ± SEM (*N* = 3) obtained using duplicate aliquots containing 9.5 μg protein (juveniles) and 10.7 μg protein (adults) from three different gill homogenates. Activity was assayed at 25°C in 50 mmol L^−1^ triethanolamine buffer (pH 7.5), containing 2 mmol L^−1^ ATP, 5 mmol L^−1^ MgCl_2_, 1.0 mmol L^−1^ NAD^+^, 0.5 mmol L^−1^ sodium phosphate, 1.0 mmol L^−1^ G3P, 150 μg GAPDH (12 U), 20 μg PGK (9 U) and NaCl (50 mmol L^−1^ for juveniles and 20 mmol L^−1^ for adults), in a final volume of 1 mL. A- Juveniles. Inset: Variation in K_0.5_ with K^+^ concentration. B- Adults. Variation in V_M_ (inset a) and K_0.5_ (inset b) with K^+^ concentration. K^+^ concentration (•) none, (○) 0.4 mmol L^−1^, (□) 0.5 mmol L^−1^, (▪) 2 mmol L^−1^, (▵) 5 mmol L^−1^, (▴) 10 mmol L^−1^, (▹) 20 mmol L^−1^.

The effect of K^+^ on NH_4_^+^-stimulated gill (Na^+^, K^+^)-ATPase activity in juvenile and adult *M. amazonicum* is shown in Fig. 7. Under saturating ATP (2 mmol L^−1^), Na^+^ (50 mmol L^−1^) and Mg^2+^ (5 mmol L^−1^) concentrations, without K^+^, stimulation of juvenile gill microsomal (Na^+^, K^+^)-ATPase activity by NH_4_^+^ (from 10^−5^ mol L^−1^ to 5×10^−2^ mol L^−1^) reached a maximum rate of 205.9±2.2 nmol Pi min^−1^ mg^−1^ with K_0.5_ = 1.9±0.2 mmol L^−1^, following Michaelis-Menten kinetics (Fig. 7A and Table 1). Modulation by NH_4_^+^ of (Na^+^, K^+^)-ATPase activity at fixed K^+^ concentrations (0.4 mmol L^−1^ to 20 mmol L^−1^) revealed no synergistic stimulation; the maximum rate reached 219.7±1.0 nmol Pi min^−1^ mg^−1^ (Table 1). However, K_0.5_ decreased 2-fold with increasing K^+^ concentrations (inset to Fig. 7A and Table 1). For 2 mmol L^−1^ K^+^ and 1 mmol L^−1^ NH_4_^+^ ([K^+^]/[NH_4_^+^]  = 2) (Na^+^, K^+^)-ATPase activity is ≈150 nmol Pi min^−1^ mg^−1^ (see Fig. 5A and 7A) indicating that the ammonium-potassium-enzyme complex is similar in both cases, independently of stimulation by either K^+^ or NH_4_^+^ at fixed NH_4_^+^ or K^+^ concentrations. The activity curves converge to similar maximum rates, as also seen for NH_4_^+^ stimulation at fixed K^+^ concentrations (Fig. 5A). This indicates that K^+^ and NH_4_^+^ bind to two different but kinetically equivalent sites.

Adult gill (Na^+^, K^+^)-ATPase activity was also stimulated by NH_4_^+^ from 10^−5^ mol L^−1^ to 5×10^−2^ mol L^−1^ (Fig. 7B). Under saturating ATP (2 mmol L^−1^), Na^+^ (20 mmol L^−1^) and Mg^2+^ (5 mmol L^−1^) concentrations, without K^+^, stimulation reached a maximum rate of 193.4±1.4 nmol Pi min^−1^ mg^−1^ with K_0.5_ = 4.8±0.3 mmol L^−1^, obeying cooperative kinetics (Fig. 7B and Table 1). At fixed K^+^ concentrations (0.5 mmol L^−1^ to 20 mmol L^−1^) stimulation of (Na^+^, K^+^)-ATPase activity was synergistic (≈36%) when the enzyme was fully saturated with NH_4_^+^. In contrast to stimulation by K^+^ at fixed NH_4_^+^ concentrations, enzyme catalytic efficiency (V_M_/K_0.5_) was greater in juveniles, independently of either ion concentration (Table 1). Both V_M_ and K_0.5_ for NH_4_^+^ stimulation were modulated by K^+^ concentration (insets **a** and **b** to Fig. 7B, respectively).

### Effect of inhibitors on juvenile and adult gill (Na^+^, K^+^)-ATPase

The effects of various inhibitors on gill total ATPase activity in juvenile and adult *M. amazonicum* are provided in Table 2. Total ATPase activity in the juvenile stage decreased from 221.6±3.2 nmol Pi min^−1^ mg^−1^ to 84.1±1.5 nmol Pi min^−1^ mg^−1^ with ouabain, suggesting ≈40% activity owing to ATPases other than the (Na^+^, K^+^)-ATPase. In the adult, these ATPases represent ≈20% of total activity. The relative proportion of different P-type ATPases was lower overall in the adult gill microsomal preparation, and Na^+^-stimulated ATPase was 10-fold greater in juveniles.

**Table 2:** Effect of various inhibitors on ATPase activity in gill microsomes from juvenile and adult *M. amazonicum.*

Inhibitor	V_M_ (nmol Pi min^−1^ mg^−1^)		% V_M_		% specific ATPase		ATPase likely present
	Juvenile	Adult	Juvenile	Adult	Juvenile	Adult	
No inhibitor (Control)	221.6±3.2	157.3±4.7	100	100	-	-	-
Ouabain (3 mmol L^−1^)	84.1±1.5	33.4±1.0	37.9	21.2	62.0±1.8	78.7±1.5*	Na^+^, K^+^-
Ouabain (3 mmol L^−1^) + DMSO (20 µL)	87.1±0.4	34.0±0.9	39.3	21.6	60.7±0.7	78.3±2.1*	-
Ouabain (3 mmol L^−1^) + Ethanol (20 µL)	83.5±0.4	32.8±1.0	37.7	20.8	62.3±0.9	79.1±1.9*	-
Ouabain (3 mmol L^−1^) + Orthovanadate (0.03 mmol L^−1^)	64.2±1.6	19.9±0.6	29.0	12.6	3.4±0.7	1.8±0.3	P-
Ouabain (3 mmol L^−1^) + Oligomycin (1 µg mL^−1^)	41.3±1.0	13.5±0.4	18.6	8.6	7.4±0.9	2.7±0.4*	F_0_F_1_-
Ouabain (3 mmol L^−1^) + Thapsigargin (0.5 µmol L^−1^)	45.9±1.1	10.3±0.3	20.7	6.5	6.5±0.6	3.1±0.5*	Ca^2+^-
Ouabain (3 mmol L^−1^) + Bafilomycin (0.4 µmol L^−1^	53.5±1.3	13.0±0.4	24.1	8.3	5.3±0.8	2.8±0.5*	V(H^+^)-
Ouabain (3 mmol L^−1^) + Ethacrynic Acid (2 mmol L^−1^)	7.6±0.2	24.7±0.7	3.4	15.7	13.1±1.3	1.2±0.4*	Na^+^- or K^+^-
Ouabain (3 mmol L^−1^) + Theophylline (5 mmol L^−1^)	29.0±0.7	12.7±0.4	13.1	8.1	9.5±1.1	2.8±0.4*	Neutral phosphatases
Ouabain (3 mmol L^−1^) + Aurovertin (0.1 mmol L^−1^)	20.0±0.5	13.0±0.4	9.0	8.3	11.0±1.0	2.8±0.6*	F_0_F_1_-

Assays were performed continuously at 25°C in 50 mmol L^−1^ HEPES buffer, pH 7.5, containing 2 mmol L^−1^ ATP, 5 mmol L^−1^ MgCl_2_, 20 mmol L^−1^ KCl and 50 mmol L^−1^ NaCl for juveniles or 20 mmol L^−1^ NaCl for adults, in a final volume of 1.0 mL. Data are the mean ± SD from three (N = 3) different microsomal preparations. Oligomycin was prepared in ethanol. Bafilomycin and thapsigargin were prepared in dimethylsulfoxide.

*Significantly different from respective value for juvenile (P≤0.05).

## Discussion

This study of (Na^+^, K^+^)-ATPase activity in zoea I, decapodid III, juvenile and adult *M. amazonicum* discloses two important findings. Firstly, K^+^ (or NH_4_^+^) modulates stimulation of (Na^+^, K^+^)-ATPase activity by NH_4_^+^ (or K^+^). Secondly, modulation of the gill enzyme's kinetic characteristics is distinctly different in juvenile and adult shrimps. K^+^ and NH_4_^+^ bind to two distinct but equivalent sites on the juvenile enzyme molecule resulting in minor stimulation, a novel finding. In the adult enzyme, each ion binds to its own specific site, providing considerable synergistic stimulation (≈50%) of (Na^+^, K^+^)-ATPase activity. The enzyme is restricted to the intralamellar septum in juvenile and adult gill lamellae, and Western blot analyses reveal just a single immunoreactive band, suggesting a sole α-subunit isoform, distributed into different density membrane fractions independently of ontogenetic stage.

Band diffusion may have resulted from the very different protein concentrations used in the two methods (SDS-PAGE and Western blot) and likely does not indicate the presence of enzyme isoforms. Differently from the biphasic inhibition curve seen in *Dilocarcinus pagei*, for example [81], our ouabain inhibition studies show a single titration curve [70] corroborating this interpretation. Further, band diffusion may be stage-specific and dependent on native enzyme protein concentration, or the enzyme may be phosphorylated to different degrees in the different stages [20].

Ion regulatory studies in freshwater crustaceans are limited mainly to large crabs, shrimps and crayfish, convenient for *in vivo* and *in vitro* experiments [30], [82]. Few studies have correlated the salinity tolerance of early ontogenetic stages with osmoregulatory capability, and there is a lack of information on the kinetic characteristics of the transporters involved [22], [50]–[53], [57]. Recently, we provided a thorough investigation of stimulation by ATP, Mg^2+^, Na^+^, K^+^ and NH_4_^+^, separately, and inhibition by ouabain of (Na^+^, K^+^)**-**ATPase activity in different ontogenetic stages of *M. amazonicum* [70].

The zoea I and decapodid III enzymes are synergistically stimulated by K^+^ at fixed NH_4_^+^ concentrations. However, these data should be regarded with caution since the (Na^+^, K^+^)-ATPase activity derives from whole larvae and not gill tissue specifically. (Na^+^, K^+^)-ATPase activity in homogenates of whole adults represents 40% of total gill (Na^+^, K^+^)-ATPase activity [70] and is comparable to *Palaemonetes argentinus* [57]. The ≈90% synergistic stimulation of (Na^+^, K^+^)-ATPase activity by K^+^ at 30 mmol L^−1^ NH_4_^+^ seen in zoea I may constitute part of an osmo-protective mechanism since zoea I is strongly euryhaline and can survive well at salinities ranging from 0 to 28‰ salinity [63]. The embryo, protected by the egg membranes suddenly eclodes into fresh water as a small, free-swimming zoea that must confront a severe osmotic challenge [83]. Whether *M. amazonicum* zoeae I hatch naturally into fresh or brackish water is not known. However, our findings suggest a role for the (Na^+^, K^+^)-ATPase in larval osmoregulation since the protein profiles for *whole* decapodid III and juvenile and adult *gill* homogenates are identical. Given that (Na^+^, K^+^)-ATPase activity is mainly concentrated in specialized gill ionocytes, and that despite the lack of functional gills, euryhaline decapod crustaceans hyper-osmoregulate on hatching, ion-transporting cells are likely located elsewhere (e. g., branchiostegite or epidermal epithelium in general) during these early ontogenetic stages [57], [84]. To illustrate, in *P. argentinus*, (Na^+^, K^+^)-ATPase activity appears to underpin embryonic osmoregulatory ability since the high (Na^+^, K^+^)-ATPase activity found close to hatching correlates with the functioning of osmoregulatory structures during late embryogenesis [57].

The present data showing variation in K_0.5_ values and in catalytic efficiency (V_M_/K_0.5_) suggest that each ion modulates activity of the other. V_M_/K_0.5_ is greater in adults than in juveniles independently of K^+^ (or NH_4_^+^) concentration. However, at higher K^+^ (or NH_4_^+^) concentrations, the juvenile enzyme is insensitive to NH_4_^+^ (or K^+^) over a wide concentration range while the adult enzyme is not. Differences in K_0.5_ values are also diagnostic. We first described synergistic stimulation of gill (Na^+^, K^+^)-ATPase activity by K^+^ and NH_4_^+^ in the blue crab *Callinectes danae* [44], [45] and, except for the freshwater crab *Dilocarcinus pagei* [81], species-specific synergistic stimulation by NH_4_^+^ plus K^+^ occurs in various crustaceans [66], [85]–[90]. The K_0.5_ for NH_4_^+^ stimulation of crustacean gill (Na^+^, K^+^)-ATPase activity is 3 to 8-fold greater than that for K^+^ [44], [66], [81], [85], [87]–[91]. The similar K_M_ values for K^+^ or NH_4_^+^ stimulation of the juvenile gill enzyme may reflect a protective mechanism against toxic NH_4_^+^ accumulation. Firstly, the similar K_0.5_ values for K^+^ modulation in the presence of NH_4_^+^, may assure K^+^ transport at elevated hemolymph NH_4_^+^ concentrations, preserving adequate intracellular K^+^ titers [92]. Secondly, the two-fold decrease in K_0.5_ values, estimated for NH_4_^+^ modulation in the presence of K^+^, may furnish a rapid response to increased hemolymph NH_4_^+^ concentration, likewise a protective response to NH_4_^+^ accumulation. The cooperative effects seen for stimulation by either K^+^ or NH_4_^+^ at fixed NH_4_^+^ or K^+^ concentrations, in contrast to the Michaelis-Menten behavior seen for stimulation of the juvenile enzyme by K^+^ or NH_4_^+^ alone, also may contribute to this protective mechanism.

The present findings suggest that synergistic stimulation by K^+^ plus NH_4_^+^ of *M. amazonicum* gill (Na^+^, K^+^)-ATPase activity is species- and stage-specific, and may underpin the active excretion of nitrogenous compounds by “extra-pumping” activity by the gill (Na^+^, K^+^)-ATPase [70]. Maximum stimulation of the juvenile enzyme by K^+^ (≈180 nmol Pi min^−1^ mg^−1^) is slightly less than that by both K^+^ and NH_4_^+^. Independently of NH_4_^+^ concentration, maximum rate is ≈205 nmol Pi min^−1^ mg^−1^ (see Fig. 5A), very similar to that for NH_4_^+^ alone (see Fig. 7A). This slight stimulation by K^+^ plus NH_4_^+^ derives from the fact that each ion occupies its respective separate site, and that, while distinct, both sites are equivalent. The similar K_0.5_ values for K^+^ and NH_4_^+^ stimulation corroborate this equivalent multisite hypothesis. In contrast, adult enzyme activity is synergistically stimulated ≈50% by K^+^ (or NH_4_^+^) under saturating NH_4_^+^ (or K^+^) concentrations, similar to the gill enzyme from *Callinectes danae* [44], *Clibanarius vittatus* acclimated to high salinity [85] and *Macrobrachium rosenbergii* [86]. This additional increase in maximum rate by K^+^ plus NH_4_^+^ suggests that the adult enzyme exhibits two different binding sites: one specific for K^+^ and the other specific for NH_4_^+^. In the presence of NH_4_^+^ both sites are occupied and (Na^+^, K^+^)-ATPase activity is stimulated to values greater than for K^+^ alone. However, increasing K^+^ concentrations displace bound NH_4_^+^ from the K^+^ binding site, and the consequent binding of K^+^ to its own site stimulates enzyme activity even further.

Both NH_3_ and NH_4_^+^ can exert toxic effects by altering cytosolic or intraorganelle pH [93], [94]. Exposure to ambient ammonia is lethal at low concentrations in crustaceans (≈1.5 mM [95]). While ammonia exists predominantly in the ionic form at physiological pH, around 2% of total ammonia is non-ionic and can diffuse readily across phospholipid membrane bilayers owing to its higher elevated lipid solubility or down a partial pressure gradient [95], [96].

The transport of toxic ammonia across gill epithelia is not fully understood, although some models are available [95], [97]. The (Na^+^, K^+^)-ATPase in the basal membrane constitutes the driving force for NH_4_^+^ transport into the ionocytes. A second NH_4_^+^-binding site that appears when the enzyme is fully saturated by K^+^ represents an additional, magnesium-inhibitable pumping force for NH_4_^+^ transport ([44], [45] and present data). Further, Cs^+^-sensitive K^+^ channels in the basal membrane [28] that do not discriminate between K^+^ and NH_4_^+^ may translocate NH_4_^+^ from the hemolymph into the cytosol [92], [97], [98]. Diffusion of NH_3_ from the hemolymph into the ionocytes also contributes to cytoplasmic NH_3_ that moves into intracellular vesicles either by diffusion or the rhesus-like ammonia transporter; a V(H^+^)-ATPase proton pump acidifies the vesicular interior, forming NH_4_^+^. An amiloride-sensitive Na^+^/NH_4_^+^(H^+^) transporter in the apical membrane, and amiloride-sensitive cation permeable structures in the cuticle may provide diffusive NH_4_^+^ efflux to the external medium. NH_4_^+^ is also extruded to the subcuticular space via an exocytotic mechanism [95], [97].

The V(H^+^)-type proton pump plays a significant role in ion uptake across the gill epithelia of freshwater-tolerant crustaceans [30], [32], [99]–[102] and has been partially kinetically characterized in adult *M. amazonicum* gills [39]. V-ATPase specific activity is ≈54 nmol Pi min^−1^ mg^−1^ in the juvenile gill microsomal fraction and 13 nmol Pi min^−1^ mg^−1^ in adult *M. amazonicum* (ca., 23 nmol Pi min^−1^ mg^−1^ [39]). The putative Na^+^/H^+^(NH_4_^+^) antiporter in the pillar cell flange apical membranes [35], [39] may contribute proportionally more to total Na^+^ uptake in adult compared to juvenile *M. amazonicum*, which appear to depend more on V(H^+^)-ATPase-dependent Na^+^ uptake [32].

The presence of a gill (Na^+^, K^+^)-ATPase showing specific kinetic characteristics that favor synergistic stimulation by NH_4_^+^ plus K^+^ may be related to different epithelial permeabilities in the different ontogenetic stages of *M. amazonicum.* Zoea I and decapodid III are usually found in moderately saline waters while juveniles and adults inhabit fresh water. Reduced body surface permeabilities likely have accompanied the occupation of brackish and freshwater habitats by marine species: to illustrate, gills of the marine crab *Cancer pagurus* show high permeabilities and constitute little or no barrier to NH_4_^+^ influx; in contrast, freshwater-adapted *Eriocheir sinensis* gills exhibit a 63-fold lower ionic permeability, which may reduce passive NH_4_^+^ influx [103]. Active ammonia excretion by *C. pagurus* is two-fold greater than in *E. sinensis*, reflecting its leaky epithelium, suggesting that an efficient mechanism of active ammonia excretion compensates for ammonia influx [103].

Elevated external ammonia may cause substitution of K^+^ by NH_4_^+^, leading to a decrease in intracellular K^+^ [92], however acute exposure of the estuarine crab *Neohelice ( = Chasmagnathus) granulata* to ammonia reveals hemolymph ammonia to be less than ambient ammonia [43], suggesting a mechanism for NH_4_^+^ excretion against its gradient [96], [103]. The active excretion of NH_4_^+^ across the gill epithelium against elevated external ammonia concentrations in the crabs *Carcinus maenas, Cancer pagurus* and *Eriocheir sinensis* [96], [103] corroborates the notion that the (Na^+^, K^+^)-ATPase is directly involved in the translocation of NH_4_^+^ from the hemolymph. An additional, magnesium-inhibitable pumping force for NH_4_^+^ extrusion, represented by a second NH_4_^+^-binding site, appears when the pump is fully saturated by K^+^ [45]. *Carcinus maenas* exposed to high NH_4_^+^ titers (2–3 mM) maintains low hemolymph NH_4_^+^ concentrations (≈100 μM) [103]. These findings suggest that most ammonia is regulated by transport proteins [93].

Some palaemonids like *M. rosenbergii* and *M. olfersi* exhibit accelerated oxidative deamination of free amino acids, which results in higher hemolymph ammonia concentrations and increased ammonia excretion rates [104]. Hemolymph NH_4_^+^, transported into the gill ionocyte cytosol by the basal (Na^+^, K^+^)-ATPase, can be exchanged for Na^+^ via an apical Na^+^/NH_4_^+^ antiporter, contributing to Na^+^ uptake [105]–[107]. This deamination mechanism may operate in adult *M. amazonicum* and, as proposed for *M. olfersi* [87], synergistic stimulation of the gill (Na^+^,K^+^)-ATPase by NH_4_^+^ plus K^+^ may constitute a valuable physiological adaptation, coupling NH_4_^+^ excretion and Na^+^ uptake in dilute media. However, cation and NH_4_^+^ fluxes across the gill cuticle of *Carcinus maenas* are inhibited by amiloride [97], [108]; thus, findings on Na^+^-dependent NH_4_^+^ transport should be interpreted cautiously since Na^+^ uptake and ammonia excretion may not be directly linked given this limiting cuticular component [95].

Concluding, we have already shown that the crustacean gill (Na^+^, K^+^)-ATPase exhibits species-specific synergistic stimulation by NH_4_^+^ plus K^+^ [44], [45], [66], [85]–[90]. Our current findings for *M. amazonicum* demonstrate changes during ontogeny, suggesting that the kinetic behavior of the gill (Na^+^, K^+^)-ATPase may be both species- and stage-specific, possibly correlating with the biochemical adjustment of each ontogenetic stage to the optimal salinity found in its natural environment. Further, sensitivity to NH_4_^+^ decreases during the ontogeny of *M. amazonicum*, which together with the synergistic stimulation by NH_4_^+^ plus K^+^ seen in adults, may underlie a novel regulatory mechanism for the crustacean (Na^+^, K^+^)-ATPase.
